# Child Brides in Africa: A Narrative Review on Effects, Gaps, and Interventions

**DOI:** 10.1002/puh2.70217

**Published:** 2026-04-27

**Authors:** Olaoye Damilola Quazeem, Onaho Pascal Oghenekaro, Durojaye Aishat Bisoye, Okoro Chidinma Peace, Akindele Folasade Diana, Aina Jesutosin Oluwadara, Dibia Ermesto Oluwafemi

**Affiliations:** ^1^ Aces Africa Serrekunda Western The Gambia; ^2^ APIN Public Health Initiative Abuja Nigeria; ^3^ University College Hospital Ibadan Nigeria; ^4^ University of Ibadan Ibadan Nigeria

**Keywords:** Africa, child marriage, cultural norms, gender equality, Nigeria, sub‐Saharan Africa, Sustainable Development Goals

## Abstract

Child marriage, defined as the union of a child under 18 years with an adult or another child, remains a pervasive issue globally, particularly in sub‐Saharan Africa. Despite international efforts to eradicate this practice, approximately 15 million girls marry before 18 annually, infringing on their basic human rights and leading to severe health, educational, and social consequences. This narrative review focuses on the high prevalence and complex causes of child marriage in sub‐Saharan Africa, especially in Nigeria, Mali, Guinea, Chad, and Niger, where cultural, economic, and societal factors sustain this harmful practice. Using databases such as Medline, PubMed, PubMed Central, and Google Scholar, alongside grey literature, to understand the prevalence, causes, and impacts of child marriage in Africa, findings highlight that child marriage leads to significant health risks, including increased maternal and infant mortality, mental health issues, and limited educational and economic opportunities for young girls. The review also examines various interventions, including legal reforms, educational programs, economic incentives, and health initiatives, that have been implemented to address this issue. Successful case studies from Ethiopia and Nigeria illustrate the potential of targeted interventions to reduce the prevalence of child marriage and improve the lives of young girls. The study underscores the urgent need for comprehensive strategies that address the root causes of child marriage and promote gender equality. Recommendations include strengthening legal frameworks, increasing access to education and economic opportunities for girls, and enhancing community awareness and engagement to shift cultural norms. By highlighting the multifaceted nature of child marriage and showcasing effective intervention programs, this research aims to inform policymakers, stakeholders, and communities about the critical need to eradicate this practice and safeguard the rights and well‐being of young African girls.

## Graphical abstract

Child marriage, defined as union before age 18, affects millions of girls across sub‐Saharan Africa, with Niger, Chad, Mali, Guinea, and Nigeria recording prevalence rates of 40–76%. This review synthesises evidence on the prevalence, sociocultural and economic drivers, and health, psychological, and social consequences of child marriage in Africa. Evidence‐based interventions; legal reforms, educational incentives, economic transfers, and health programmes are assessed, and recommendations for policy and practice are proposed.

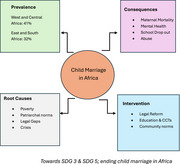

## 1 Introduction

Child marriage has been defined as a formal or informal union where at least one partner is under the age of 18 [1]. It remains one of the most persistent violations of human rights and gender equality globally [1, 2]. Despite international commitments to eradicate the practice, it continues to affect millions of girls every year. Recent global estimates suggest that one in five young women (approximately 19%) was married before their eighteenth birthday, with sub‐Saharan Africa recording the world's highest prevalence and slowest rate of decline [3, 4]. In this region, nearly 120 million girls and women alive today were married as children, and projections indicate that if current trends persist, Africa will become home to more than half of the world's child brides by 2050 [5].

The gravity of this issue extends beyond statistics; it reflects the structural inequalities, patriarchal norms, and socioeconomic vulnerabilities that deprive girls of their rights to education, autonomy, and health [6, 7]. In Western and Central Africa, 6 of the 10 countries with the highest rates of child marriage globally, Niger, Chad, Mali, Guinea, the Central African Republic, and Nigeria, record prevalence rates ranging between 40% and 76% [4, 8]. For example, Nigeria, Africa's most populous country, has one of the largest absolute numbers of child brides, with an estimated 22 million girls married before the age of 18 [9]. The persistence of this practice underscores the complex sociocultural, economic, and legal dynamics that sustain it despite global and national policy commitments to its eradication.

Africa remains the epicenter of child marriage, not only because of its high prevalence but also because of the diversity of its cultural and religious drivers, and the significant health and economic consequences associated with the practice [10, 11]. The region faces a “triple burden” of poverty, gender inequality, and weak legal enforcement mechanisms that perpetuate harmful traditional practices. The interplay of customary law, religious norms, and economic hardship has made child marriage a deeply entrenched social institution rather than a mere violation of law [12, 13].

Although most African countries have ratified the Convention on the Rights of the Child (CRC) and the African Charter on the Rights and Welfare of the Child, the coexistence of statutory, customary, and religious laws, known as legal pluralism, has created inconsistencies in the enforcement of minimum marriage age laws [14]. For instance, while 90% of sub‐Saharan African nations set 18 years as the legal age of marriage, nearly one‐third permit exceptions through parental or judicial consent [15]. Such loopholes perpetuate the practice by legitimizing it within traditional and religious frameworks. Customary systems often prioritize family alliances and economic transactions, such as bride price, over individual rights, whereas some religious interpretations, especially within conservative Islamic and Christian communities, are used to justify early unions as moral safeguards against premarital sex [16, 17].

Similarly, although there are several country‐specific and thematic studies that have examined aspects of child marriage, there is still a fragmented understanding of how these factors interact across the African context. Many reviews have focused on either prevalence or interventions, but rarely integrate the spectrum from causes to outcomes and responses [18, 19]. Moreover, empirical data on the mental health effects, resilience of child brides, and evaluation of large‐scale interventions remain limited, particularly in low‐income and conflict‐affected countries.

This narrative review, therefore, aims to synthesize and contextualize existing evidence on child marriage in Africa, providing a holistic understanding of its prevalence, underlying determinants, and health and social consequences. It also reviews evidence‐based interventions and identifies knowledge gaps that can inform future research, policy, and programming. Specifically, the objectives of this study are to:

1. Examine the prevalence and patterns of child marriage across African regions.
2. Explore the socioeconomic, cultural, religious, and legal determinants of child marriage.
3. Analyze the health, psychological, and social consequences of child marriage for girls and their communities.
4. Assess the effectiveness of interventions aimed at reducing child marriage and promoting gender equality.

By consolidating findings from diverse sources, this review seeks to contribute to a more nuanced understanding of child marriage as a multidimensional public health and human rights issue in Africa. The study highlights the pressing need for culturally grounded and legally harmonized strategies that empower girls, challenge patriarchal norms, and ensure accountability in policy implementation to achieve the Sustainable Development Goals (SDGs), particularly SDG 3 (good health and well‐being) and SDG 5 (gender equality).

## 2 Materials and Methods

This study employed a narrative review design, which is appropriate for synthesizing and interpreting existing knowledge on complex public health and social issues such as child marriage. Unlike systematic reviews, which adhere to rigid inclusion criteria and quantitative meta‐analyses, narrative reviews emphasize critical interpretation, thematic synthesis, and contextual integration of diverse sources of evidence [20, 21, 22]. This approach allows for a more holistic understanding of multidimensional phenomena, particularly those influenced by cultural, legal, and sociopolitical contexts, as is the case with child marriage in Africa.

### 2.1 Search Strategy

A comprehensive search of both peer‐reviewed and grey literature was conducted across multiple electronic databases, PubMed, PubMed Central, Medline, and Google Scholar, covering all studies published up to March 2025. To ensure inclusivity and relevance, supplementary searches were also conducted through the websites of major international agencies and nongovernmental organizations such as UNICEF, UNFPA, WHO, Girls Not Brides, and the World Bank.

The following combination of keywords and Boolean operators was used:

(“child marriage” OR “early marriage” OR “child bride” OR “forced marriage”) AND (“Africa” OR “Sub‐Saharan Africa” OR “West Africa” OR “East Africa” OR “Central Africa”) AND (“health outcomes” OR “gender inequality” OR “education” OR “intervention” OR “policy response”).

The reference lists of retrieved studies and relevant reviews were also hand‐searched to identify additional eligible publications not captured in the database search.

### 2.2 Eligibility Criteria

Inclusion criteria were developed to ensure that selected studies directly contributed to the review's objectives. Studies were included if they:

- Focused on child marriage or early marriage within African countries;
- Examined prevalence, determinants, consequences, or interventions related to the practice;
- Were published in English;
- Represented peer‐reviewed research, policy reports, or credible grey literature.

Studies were excluded if they:

- Focused primarily on non‐African contexts;
- Were opinion pieces without empirical or policy relevance;
- Contained duplicated data or lacked methodological detail.

No restrictions were placed on the year of publication to capture the full historical and contemporary scope of literature addressing child marriage in Africa.

### 2.3 Screening and Data Management

Seven independent researchers, each trained in sexual and reproductive health research, participated in the screening and data extraction process. A total of 33 articles were retrieved and all retrieved articles were imported into EndNote X8 for reference management and de‐duplication. Screening proceeded in two stages:

1. Title and abstract review to assess preliminary relevance;
2. Full‐text review to confirm eligibility.

Disagreements between reviewers were resolved through discussion and consensus. For each included study, data were extracted using a standardized template covering the following domains: author, year, study location, design, sample characteristics, key findings, and thematic focus (e.g., prevalence, drivers, health consequences, or interventions).

### 2.4 Data Synthesis and Analytical Approach

A narrative Synthesis approach was adopted, consistent with the methodological reasoning advanced by Greenhalgh et al. [20], who challenged the assumed superiority of systematic reviews and informed by the review typology established by Grant and Boot [22] and the reporting guidance provided by Ferrari [21]. Evidence was synthesized inductively, grouping findings under major themes consistent with the review objectives: (i) prevalence and root causes, (ii) consequences, and (iii) interventions.

Within each theme, patterns and relationships were identified across studies, allowing for comparison of similarities and differences among countries and subregions. Grey literature, such as government reports, NGO evaluations, and United Nations publications, was integrated to complement academic sources, particularly where data gaps existed in peer‐reviewed studies.

To enhance the credibility of the synthesis, all extracted information underwent cross‐validation among reviewers. Particular attention was given to highlighting methodological gaps, inconsistencies in prevalence data, and contextual factors (legal, cultural, and economic) that influenced outcomes. The resulting synthesis thus presents a holistic view of the burden, determinants, and responses to child marriage across Africa.

### 2.5 Ethical Considerations

This review analyzed secondary data and published literature; therefore, ethical approval was not required. However, all data sources were appropriately cited, and intellectual property rights were respected in line with academic publishing standards.

### 2.6 The Prevalence and Root Causes of Child Marriage: Exploring the Social, Economic, and Cultural Factors Affecting the Incidence of Child Marriage

Child marriage remains a widespread and deeply complex phenomenon with highly varied estimates depending on region, methodology, and data source. The review by Tembo notes that about 7.3 million girls marry before 18 in low‐ and middle‐income countries, a violation of their fundamental human rights with far‐reaching consequences on the development of young girls [23]. To provide a more nuanced perspective, recent systematic reviews suggest that among surveyed settings, the incidence may vary from approximately 1.8% to 90.8%, influenced by factors such as locality, urban or rural settings, and age demographics in individual studies [7].

According to a 2017 UNFPA–UNICEF report, the prevalence of child marriage in West and Central Africa stands at 41%, accounting for nearly 60 million girls [24]. Similarly, UNICEF estimates that in Eastern and Southern Africa, more than 50 million women were married before the age of 18, with approximately 32% young women entering marriage in childhood [25]. In the Middle East and North Africa region, prevalence ranges from 2% in Tunisia to 34% in Sudan, averaging at 13.3% [26]. Although the practice of child marriage is generally declining across Africa, the pace of reduction remains uneven; some countries have achieved sustained progress, whereas others have stagnated or regressed. Projections nonetheless indicate that, without accelerated interventions, an additional 20.8 million girls in the region could become child brides by 2050 [25].

Child marriage is driven by an interplay of cultural, social, religious, and economic factors that are deeply interwoven. Studies indicate that socioeconomic pressures often play the most significant role in the prevalence of child marriage [27]. Key drivers include poverty and economic hardship, limited alternatives for girls, such as lack of education or livelihood opportunities, gender‐inequitable social norms, and concerns about premarital sexuality, all underpinned by girls’ limited agency and decision‐making power [28]. These factors rarely act in isolation; instead, they reinforce one another in sustaining the practice [27].

Culturally, in many African societies, patriarchal family structures perpetuate child marriage by placing control over marriage decisions in the hands of male family members. This patriarchal influence often determines the timing of the marriage, arrangement, and spousal selection with little input from the girl herself [13]. Additionally, deeply entrenched gender inequality and societal expectations around women's roles primarily as wives and mothers limit their autonomy. Research from Ethiopia, for example, finds that women's low social status, rooted in patriarchy, the “feminization” of poverty, and low educational attainment, play a crucial role in sustaining high rates of early marriage [29]. Moreover, a long‐standing cultural belief is that marrying girls early allows them to maximize their fertility potential, underlying the notion that a younger bride will have more reproductive years and thereby bear more children for the family [21]. This belief can be especially influential in patriarchal settings where a woman's value is tied to childbearing.

Another factor includes concerns about purity and honuor. For the sake of preserving sexual purity before marriage, which is held in high regard in typical African homes, some girls are married off early, before their pubertal years, to avoid promiscuity and being unfit to marry culturally [16]. In many communities, premarital chastity is tied directly to family honuor and a girl's marriageability [29]. Parents often fear that delaying marriage will increase the risk of their daughters engaging in “immoral” behaviour, becoming promiscuous, or getting pregnant out of wedlock, any of which would be seen as a disgrace to the family. For instance, a cross‐sectional study reports that marrying a girl early is considered desirable because it protects the purity of the paternal line, thereby safeguarding lineage [30].

In certain social contexts, notably war‐torn regions and humanitarian crisis settings, spikes in child marriage are observed as a coping or protective mechanism. Parents living amid conflict or in refugee settlements often worry intensely about the security of adolescent daughters. Marrying them off is seen as providing stability, protection, and economic security in an otherwise perilous environment [31]. In these situations, families may genuinely believe their daughters will be safer from sexual violence if married early, since unaccompanied girls in conflict zones or camps face high risks of harassment and assault [17]. Unfortunately, while early marriage in such cases is intended to shield girls from external harm, it often exposes them to other dangers, including domestic violence, marital rape, and severe mental health stress, essentially trading one form of risk for another [31]. Family dysfunction or instability can further exacerbate the likelihood of child marriage in these environments. The breakdown of traditional support systems, for example, the death or absence of a parent, leaves many girls more vulnerable to being married off. Evidence from humanitarian contexts shows that an orphaned girl's chances of early marriage increase when extended family members struggle to support her basic needs [26]. In other cases, girls from abusive or volatile families may themselves view marriage as an escape, not fully recognizing the harms early marriage can bring.

Place of nurture, otherwise referred to as the environment the female child grows up in, determines the degree of exposure to the child's marriage practice and eventual outcome if no intervention occurs. Rural, suburban, and slum areas tend to have significantly higher rates of child marriage compared to urban and metropolitan areas [18]. These areas also typically have reduced access to education and economic opportunities for girls, creating a situation where marriage may appear to be the only viable path. Recent reviews have confirmed that girls living in rural areas, as well as those in less developed communities, face higher odds of experiencing child marriage [7]. By contrast, girls in urban centers marry later on average, likely due to better access to schooling, greater exposure to modern values that discourage child marriage, and increased autonomy for young women. As a result, even within the same country, the prevalence of child brides can vary drastically based on the regions [29].

Religious beliefs are often cited as a contributing factor to early marriage. The practice is not confined to a single religion, as it occurs among various religious communities, but certain conservative interpretations have explicitly or implicitly sanctioned marrying girls at a young age. For instance, some ultratraditional Islamic communities believe that marrying off a girl child early aligns with religious teachings and the supposed practices of Islamic history. They often cite permissive interpretations of scripture and historical accounts, such as the marriage of the Prophet Muhammad to Aisha, who is said to have been about 9 years old at the time [12]. However, many Muslim scholars and communities have emphatically opposed child marriage, arguing that such early unions violate Islamic principles of justice and the well‐being of children [32]. In most cases, it is often the fusion of religion with traditional culture, combined with weak legal enforcement, that allows these practices to continue [33].

Finally, poverty is widely recognized as the most pervasive root cause of child marriage. Impoverished families are far more likely to marry off their daughters early, largely because of economic necessity and incentive. Large families, in particular, often give their daughters away in exchange for a bride price [19, 20]. They see marrying off one or more daughters as a way to reduce mouths to feed, gain resources, and possibly forge beneficial ties with another family. Numerous studies across Africa confirm a strong inverse correlation between household economic status and the age at marriage: Poorer households have markedly higher rates of child brides than wealthier ones [34]. Even where other factors, cultural or religious, provide justification for early marriage, economic desperation typically underlies the decision. Tackling child marriage, therefore, ultimately requires addressing this economic dimension alongside the social and cultural drivers.

### 2.7 Consequences of Child Marriages on Girls and Their Communities: Investigating the Physical, Psychological, and Social Consequences of Underage Marriage on Girls

The consequences of child marriage on girls are extensive, affecting their general health, sexual and reproductive rights, socioeconomic well‐being, the survival of their children, and the broader prosperity of their families and communities. Most child brides are traditionally expected to become pregnant within the first year of marriage, placing them at heightened risk of miscarriages, obstetric complications, and pregnancy‐related fatalities [35]. Adolescent mothers, between the ages of 10 and 19, are significantly more likely to experience maternal mortality than women in their twenties, with early pregnancies contributing to low birth weight, preterm birth, and neonatal mortality [36]. Recent evidence confirms that early childbearing remains a leading contributor to maternal and infant morbidity in these settings [37].

Furthermore, child brides are more susceptible to domestic violence and intimate partner violence (IPV) [11]. Their young age, coupled with lower socioeconomic status and limited decision‐making power, increases vulnerability to physical, sexual, and emotional abuse [38]. Studies indicate that married adolescents often have little autonomy to refuse sex or negotiate safe sexual practices, exposing them to HIV and other sexually transmitted infections and compounding reproductive health risks [39]. In many cases, their limited access to maternal and reproductive healthcare further exacerbates these vulnerabilities, creating a cascade of interlinked health challenges [40].

Beyond physical and reproductive risks, child marriage deprives young women of the opportunity to realize their full potential as healthy and productive individuals, condemning them to poverty and constraining life choices [21]. A particular challenge arises from the fact that child brides, who themselves are still adolescents, are often ill‐prepared to assume parental responsibilities, lacking sufficient knowledge about sexual and reproductive health and child‐rearing practices [39]. This intergenerational vulnerability contributes to poor child outcomes and perpetuates cycles of disadvantage.

The psychological impact of child marriage is equally profound. Evidence demonstrates a strong link between early marriage and depression, anxiety, and emotional distress, often exacerbated by IPV [41]. The sudden transition from childhood to marital and parental responsibilities results in social isolation, loss of peer networks, and diminished agency, increasing psychological strain [42]. Early pregnancy, compounded by limited social support, can also intensify stress and contribute to suicidal thoughts or attempts [43]. Obstetric complications such as fistula, as well as early sexual exposure, further reinforce these mental health challenges [35].

Another significant consequence of child marriage is the disruption of education and social development. Girls married before 18 are frequently forced to leave school, terminating their educational trajectory [21, 44]. This disruption impairs literacy and the acquisition of life skills, limiting future employment opportunities and economic independence [38]. Studies show that children of adolescent mothers are also more likely to experience educational disadvantages, perpetuating intergenerational cycles of low schooling and reduced socioeconomic mobility [45]. The loss of education, coupled with limited autonomy, prevents girls from participating meaningfully in household or community decision‐making, reinforcing gender inequality.

Economically, child marriage imposes long‐term constraints at both the individual and household level. With reduced education and limited vocational skills, child brides are often excluded from formal labor markets and confined to domestic or informal work [10, 27]. As a result, they depend financially on their husbands, limiting their capacity to influence family resources or escape abusive environments [43]. Additionally, research suggests that early marriage reduces expected lifetime earnings, with some studies estimating a 9% loss in potential wages due to curtailed schooling and reduced productivity [46]. At the household level, larger families resulting from early and frequent childbearing exacerbate poverty, while at the societal level, aggregate human capital losses hinder economic development [19, 20]. In contrast, ending child marriage has been shown to yield measurable economic gains, including increased female labor participation, reduced fertility‐related health costs, and improved household welfare [45]. This underscores the urgent need for targeted, evidence‐based policies to eliminate child marriage as a catalyst for inclusive and sustained economic growth. Addressing child marriage, therefore, is critical not only for safeguarding adolescent girls’ rights and well‐being but also for promoting healthier, more productive, and equitable communities.

### 2.8 Intervention Programs and Strategies

Although numerous initiatives aim to tackle child marriage in Africa, evidence reveals a significant gap in implemented interventions. A 2024 study by Kidman et al. [47] illustrates that although there have been interventions that have been introduced in areas with high rates of marriage, their outcomes are still mixed and lack a synergistic framework. Similarly, a 2021 systematic review identified only 15 interventions actively underway across the continent, highlighting the need for a greater focus on translating efforts into concrete and scalable actions [11]. To ensure the thriving development of societies, the life prospects of adolescents, and their overall well‐being, prioritizing the implementation of an evidence‐based approach to eradicating child marriage and teenage pregnancies is imperative [48]. Despite the evident gap in interventions, some studies and program evaluations have identified effective interventions that demonstrably delay and ultimately prevent child marriage, leading to positive outcomes for girls and communities. These successes highlight the critical role of dedicated programs in tackling this harmful practice [49]. Something to note is that studies have also shown that a concerning imbalance exists in the research conducted across different geographic regions. Approaches in areas like Southern, Northern, Western, and Middle Africa remain below expected levels, given the widespread nature of the practice [50]. This observation aligns with findings from Greene et al. [51], who noted that the scale required to effectively combat child marriage has not been adequately reflected in current evidence. Multiple interventions and approaches have been developed to prevent child marriage and to support individuals who entered marriage during childhood. These approaches include Legal and Constitutional reforms, educational interventions, economic and livelihood interventions, norms‐based interventions, and health‐centered interventions or programs.

### 2.9 Constitutional Interventions

Various interventions designed to address child marriage involve the amendment of the constitution and legal practices. Legal and constitutional reforms represent an essential pillar in national strategies to reduce child marriages, although their effectiveness depends heavily on enforcement and community acceptance. On January 20th, 2016, the Zimbabwean Constitutional Court issued a ruling that prohibited child marriage and related practices [52]. Furthermore, the court's decision prompted subsequent legislative effort to harmonize and codify marriage laws within the framework of the 2013 constitution, ensuring legal consistency and strengthening protections against child marriage [52]. Although this reform significantly strengthened the legal framework protecting children, reports indicate that implementation challenges and persistent social norms have limited the ruling's immediate impact on reducing marriage rates [52]. Nevertheless, this constitutional reform still remains an important foundation for long‐term change. Evidence from other parts of sub‐Saharan Africa reinforces this point. Maswikwa et al. found that countries with laws that consistently set the minimum age of marriage for girls at 18 or older had substantially lower rates by 40% of child marriage and adolescent births compared to those without such legal coherence [53].

Beginning in the year 2000, nine regions in Ethiopia accepted positive changes to their family laws by increasing the least acceptable age of marriage from 15 to 18 [50]. There have been mixed outcomes, with reductions in early marriage occurring primarily in regions where legal changes were supported by community engagement, awareness efforts, or broader socioeconomic interventions. In areas lacking these complementary measures, legal changes alone did not significantly alter marriage practices [50, 52].

### 2.10 Educational Interventions

Either through structural investments or incentive‐based programs, educational interventions in combating child marriages demonstrate strong evidence of reducing early marriage. Although the effectiveness varies by context, age group, and type of support provided.

By attaining higher education, women gain greater control over their reproductive lives and career paths. This is evidenced by the direct proportionality of increased female education to marrying and giving birth at older ages. Higher education rates for females have also led to reduced fertility, aligned fertility desires, personal contraceptive choices, and expanded access to non‐domestic employment opportunities [54]. Several studies and reports have discovered that there is a decline in the rate of early marriage as a result of educational incentives [54, 55, 56].

Studies evaluating specific educational incentives show more direct effects. Transfer of cash, a form of financial assistance, offers a powerful strategy to combat child marriage through two key mechanisms: providing critical educational support for girls and incentivizing school attendance by linking payments to educational milestones and delaying marriage [50]. An example includes the Research Initiative to Support the Empowerment of Girls (RISE) program in the small, undeveloped parts of Zaria, which increased school retention [57]. More robust evidence comes from a 7‐year randomized evaluation in Kenya that revealed that education subsidies significantly decreased the probability of girls and boys becoming school drop‐outs, getting married prematurely, and experiencing early pregnancy within marriage [55]. Findings from other studies also report declines in early marriage associated with educational support; however, the magnitude of impact varies, with some interventions showing strong reductions and others producing only incremental changes [54, 55, 56].

### 2.11 Economic Interventions

There is evidence that shows that economic interventions aimed at the reduction of child marriages across African countries have produced mixed effectiveness. That is, not all economic programs have consistently reduced early marriage rates even though poverty is a major driver pushing families to marry off their young daughters early. In a systematic review by Malhotra and Elnakib [19], Conditional Cash Transfers (CCT) which tie incentives such as tuition payments, stipends, or school supplies to girls’ school attendance or delayed marriage were among the most consistently effective interventions in reducing early marriage [58, 59, 60, 61, 62]. In contrast, Unconditional Cash Transfers (UCTs) showed weaker and less reliable impacts. This suggests that incentives work best when directly tied or linked to behaviors that keep girls in school.

Recognizing the value of specific resources within local communities, another economic intervention that has been carried out included adapted incentives to local context where noncash, culturally relevant assets such as livestock (chickens or goats) or cooking oil were provided annually or upon the girl reaching age 18 unmarried. These incentives improved school retention but the impact on actual marriage delay varied, with some communities demonstrating strong uptake while others showed minimal behavioral change [63]. An evaluation carried out by the population council across Burkina Faso, Ethiopia, and Tanzania compared four approaches which included community dialogues, provision of school or livelihood assets, a combined comprehensive model, and a control arm. Findings showed that economic assistance was particularly effective for older adolescents but less effective for younger girls, whose marriages are often due to other reasons that are more titled toward societal norms and culture rather than family economics [49]. This study showed that economic interventions are not sufficient when employed alone and must be paired with community norm‐change strategies in order to achieve sustained reductions in child marriage.

### 2.12 Health Interventions

Health interventions addressing reproductive, mental, and social well‐being have contributed to improvements in adolescents’ knowledge and resilience, although their direct effect on reducing early marriage still varies by program.

The Oromia Development Association project in Ethiopia, which was a school‐based initiative, demonstrated positive outcomes in knowledge, gender attitudes, and empowerment but there is limited evidence for a direct reduction in marriage rates. Nevertheless, there seems to be an association between awareness of reproductive rights and gender equality, and an improved ability of girls to negotiate or resist early marriage pressures [50, 64].

Programs focused on mental health show promising indirect effects. For example, Ethiopia's Child Resilience Program improved children's coping skills, problem‐solving abilities, and peer support. Evaluation reports revealed that participants were sometimes able to intervene in or avoid early marriage, therefore, indicating a preventive influence, although this was not the program's objectives [63, 65]. The peer support groups for young mothers in Zimbabwe, which is an example of programs targeting already married adolescents, reported gains in mental health, social connectedness, and skills development, but these primarily supported married girls rather than preventing marriage [66]. Similarly in Amhara, Ethiopia, the TESFA program, which was designed to support married adolescents, produced improvements in psychosocial well‐being and communication, although this did not directly aim to delay or reduce marriage [62]. These findings highlight that health interventions play a valuable supportive role but require integration with border economic and community‐level strategies to directly impact early marriage rates.

### 2.13 Case Studies From Different African Countries That Have Successfully Reduced Child Marriage Through Innovative Strategies

Targeting married girls in Ethiopia's Amhara region, the CARE TESFA program aimed to improve their lives and challenge the prevalence of child marriage. It equipped participants with valuable financial and reproductive health knowledge through interactive curricula delivered via peer‐based solidarity groups, fostering collective learning and empowerment [67]. TESFA, meaning “hope” in Amharic, championed a revolutionary approach: empowering married girls. Through peer solidarity groups, it offered crucial reproductive health knowledge and economic opportunities, guiding them toward a brighter future [67]. In 2013, TESFA conducted a completion evaluation of the program, which detected tangible economic, health, and social improvements in the lives of those who participated [62]. Beyond tangible results, TESFA equipped girls with valuable knowledge. From family planning and antenatal care to understanding STIs and fertility, they gained newfound control over their health and reproductive choices. Their voices also gained weight, with increased participation in household decision‐making and newfound freedom of movement [62]. TESFA's success in Ethiopia demonstrates the transformative power of empowering married girls. By replicating its approach and prioritizing interventions for those already caught in child marriage, we can chart a new course with a better and more inspiring future for young girls all over the world.

Stepping into the shoes of girls facing immense odds, in the city of Zaria, the Centre for Girls Education (CGE) birthed Pathways to Choice. This innovative program, meticulously crafted through community voices, tackled the twin challenges of child marriage and lack of education in northern Nigeria. Through mentoring girls’ clubs and financial support for school enrolment, Pathways offered vulnerable girls a safety net and a brighter future [68].

## 3 Recommendation and Conclusion

This review shows that child marriage is associated with significant adverse outcomes that span across health, psychological, educational, and economic domains. Girls who marry before the age of 18 face elevated risks of maternal morbidity and mortality, obstetric complications, IPV, and even limited autonomy in reproductive decision‐making. The review also demonstrates that early marriage is also strongly linked to school discontinuation, reduced literacy and skills acquisition, restricted labour market participation, and lower lifetime earnings. These effects also extend to further generations, contributing to poorer child health and educational outcomes and reinforcing cycles of socioeconomic disadvantages.

The multidimensional nature of the consequences of child marriage indicates a need for multisectoral and coordinated response. The evidence reviewed suggests that effective strategies should combine enforcement of existing legal frameworks with investment in girls’ education, adolescent‐responsive health services, social protection measures, and economic empowerment initiatives. There should be clear operational guidelines and accountability mechanisms which are necessary to ensure harmonized responses across sectors. Engagement of healthcare providers, legal and judicial actors, educators, community and religious leaders, policymakers, parents, and adolescents appears central to prevention and response efforts. Strengthened interministerial coordination and sustained monitoring of evidence‐informed interventions may be important for reducing prevalence and mitigating documented harms.

## Author Contributions

**Olaoye Damilola Quazeem:** conceptualization, supervision, methodology; writing – original draft and editing. **Okoro Chidinma Peace:** participated in evidence synthesis, Formal analysis and writing. **Dibia Ernesto Oluwafemi:** writing – original draft and formal analysis. **Durojaye Aishat Bisoye:** investigation, writing – original draft, writing – review and editing, resources. **Onaho Pascal Oghenekaro**: writing – original draft, writing – review and editing, investigation, resources. **Akindele Folasade Diana**: investigation, writing – original draft, resources. **Aina Jesutosin Oluwadara**: writing – original draft, investigation, resources.

## Conflicts of Interest

The authors declare no conflicts of interest.

## Data Availability

Data availability is not applicable in this narrative review.
